# Maternal Zika Virus (ZIKV) Infection following Vaginal Inoculation with ZIKV-Infected Semen in Timed-Pregnant Olive Baboons

**DOI:** 10.1128/JVI.00058-20

**Published:** 2020-05-18

**Authors:** Sunam Gurung, Hugh Nadeau, Marta Maxted, Jamie Peregrine, Darlene Reuter, Abby Norris, Rodney Edwards, Kimberly Hyatt, Krista Singleton, James F. Papin, Dean A. Myers

**Affiliations:** a Department of Obstetrics and Gynecology, University of Oklahoma Health Sciences Center, Oklahoma City, Oklahoma, USA; b Department of Pathology, University of Oklahoma Health Sciences Center, Oklahoma City, Oklahoma, USA; c Division of Comparative Medicine, University of Oklahoma Health Sciences Center, Oklahoma City, Oklahoma, USA; University of Kentucky College of Medicine

**Keywords:** Zika, baboon, fetus, flavivirus, placenta, pregnancy, primate, sexual transmission, vertical transfer, virus

## Abstract

Zika virus (ZIKV) infection is now firmly linked to congenital Zika syndrome (CZS), including fetal microcephaly. While *Aedes* species of mosquito are the primary vector for ZIKV, sexual transmission of ZIKV is a significant route of infection. ZIKV has been documented in human, mouse, and nonhuman primate (NHP) semen. It is critical to establish NHP models of the vertical transfer of ZIKV that recapitulate human pathogenesis. We hypothesized that vaginal deposition of ZIKV-infected baboon semen would lead to maternal infection and vertical transfer in the olive baboon (Papio anubis). Epidemiological studies suggest an increased rate of CZS in the Americas compared to the original link to CZS in French Polynesia; therefore, we also compared the French Polynesian (FP) ZIKV isolate to the Puerto Rican (PR) isolate. Timed-pregnant baboons (*n* = 6) were inoculated via vaginal deposition of baboon semen containing 10^6^ focus-forming units (FFU) of ZIKV (*n* = 3 for FP isolate H/PF/2013; *n* = 3 for PR isolate PRVABC59) at midgestation (86 to 95 days of gestation [dG]; term, 183 dG) on day 0 (all dams) and then at 7-day intervals through 3 weeks. Maternal blood, saliva, and cervicovaginal wash (CVW) samples were obtained. Animals were euthanized at 28 days (*n* = 5) or 39 days (*n* = 1) after the initial inoculation, and maternal/fetal tissues were collected. Viremia was achieved in 3/3 FP ZIKV-infected dams and 2/3 PR ZIKV-infected dams. ZIKV RNA was detected in CVW samples of 5/6 dams. ZIKV RNA was detected in lymph nodes but not the ovaries, uterus, cervix, or vagina in FP isolate-infected dams. ZIKV RNA was detected in lymph nodes (3/3), uterus (2/3), and vagina (2/3) in PR isolate-infected dams. Placenta, amniotic fluid, and fetal tissues were ZIKV RNA negative in the FP isolate-infected dams, whereas 2/3 PR isolate-infected dam placentas were ZIKV RNA positive. We conclude that ZIKV-infected semen is a means of ZIKV transmission during pregnancy in primates. The PR isolate appeared more capable of widespread dissemination to tissues, including reproductive tissues and placenta, than the FP isolate.

**IMPORTANCE** Zika virus remains a worldwide health threat, with outbreaks still occurring in the Americas. While mosquitos are the primary vector for the spread of the virus, sexual transmission of Zika virus is also a significant means of infection, especially in terms of passage from an infected to an uninfected partner. While sexual transmission has been documented in humans, and male-to-female transmission has been reported in mice, ours is the first study in nonhuman primates to demonstrate infection via vaginal deposition of Zika virus-infected semen. The latter is important since a recent publication indicated that human semen inhibited, in a laboratory setting, Zika virus infection of reproductive tissues. We also found that compared to the French Polynesian isolate, the Puerto Rican Zika virus isolate led to greater spread throughout the body, particularly in reproductive tissues. The American isolates of Zika virus appear to have acquired mutations that increase their efficacy.

## Abstract (precis)

Zika virus remains a worldwide health threat, with outbreaks still occurring in the Americas. While mosquitos are the primary vector for the spread of the virus, sexual transmission of Zika virus is also a significant means of infection, especially in terms of passage from an infected to an uninfected partner. While sexual transmission has been documented in humans, and male-to-female transmission has been reported in mice, ours is the first study in nonhuman primates to demonstrate infection via vaginal deposition of Zika virus-infected semen. The latter is important since a recent publication indicated that human semen inhibited, in a laboratory setting, Zika virus infection of reproductive tissues. We also found that compared to the French Polynesian isolate, the Puerto Rican Zika virus isolate led to greater spread throughout the body, particularly in reproductive tissues. The American isolates of Zika virus appear to have acquired mutations that increase their efficacy.

## INTRODUCTION

The propagation of Zika virus (ZIKV) represents a worldwide reproductive health crisis given the virus’s geographic distribution and the severity of its effect on the developing fetus. At its peak, ZIKV infection was being reported in more than 60 countries (1, 2). While ZIKV infection is usually mild or asymptomatic, with typical symptoms including fever, rash, and conjunctivitis, the impact of ZIKV on the developing fetus can be severe, including microcephaly and associated neurological damage, fetal growth restriction, intrauterine fetal demise, spontaneous abortion, as well as other congenital anomalies, now collectively termed congenital Zika syndrome (CZS). The impact of ZIKV infection on the fetus led to worldwide interest in the virus following a major outbreak in Brazil in 2015 (3).

While *Aedes* species of mosquitos (Aedes aegypti) are the primary means of ZIKV transmission, male-to-female as well as female-to-male sexual transmission of ZIKV is now well documented in the human population (4–8), and there is a greater incidence of ZIKV in females of a sexually active age (9), suggesting that sexual transmission is a causative factor. Sexual transmission in humans was first reported in 2008 (10), and from 2015 to 2018, 52 cases of sexually transmitted ZIKV infection were reported in the United States (11). Sexual transmission of ZIKV from a male to a pregnant female has also been described (12, 13). ZIKV RNA and infectious virus have been detected in human semen (up to 10^7^ copies/ml) (14), for a considerably longer time than detection in blood, saliva, and urine (6). ZIKV RNA has been detected in semen 188 days after the onset of symptoms, while infectious ZIKV has been observed in human semen up to 69 days after symptoms (8, 15). We recently demonstrated ZIKV RNA in semen in male baboons up to 41 days postinfection (dpi), which was when the study was terminated, and the persistence of ZIKV RNA in the male reproductive tissues after systemic resolution of the virus, thus confirming findings in the human population (16). Male-to-female sexual transmission of ZIKV has been observed in an interferon (IFN)-deficient (AG129) mouse model of ZIKV infection, resulting in a high degree of infection of AG129 females, including vertical transfer. In addition, vaginal ZIKV inoculation resulted in a high degree of infection in both nonpregnant and pregnant AG129 females (17, 18). Yockey et al. (19) similarly found that ZIKV replicated in the vaginal mucosa of wild-type mice and, while not leading to viremia, resulted in fetal growth restriction and infection of fetal brains, indicating that vaginal inoculation may lead to the direct transfer of the virus to the intrauterine compartment through a compromised cervical canal or via local lymphatics to the uteroplacental interface. Vaginal inoculation of nonpregnant female rhesus monkeys with ZIKV has also been achieved albeit using raw virus in a culture medium diluent rather than semen in the inoculant. A recent study suggested that human semen may actually inhibit ZIKV infectivity in an *in vitro* setting, including in human reproductive tract explants (20). Studies estimate that sexual transmission of ZIKV in the human population is responsible for a substantial number of infections and the maintenance of the virus in the human population with or without the presence of the vector. This is particularly of concern for pregnant women, as studies have shown that ZIKV is disseminated to the placenta and fetus after intravaginal infections and causes CZS in the fetus (7, 19, 21, 22). Sexual transmission is also a likely means of the global spread of the virus between landmasses.

**FIG 1:**
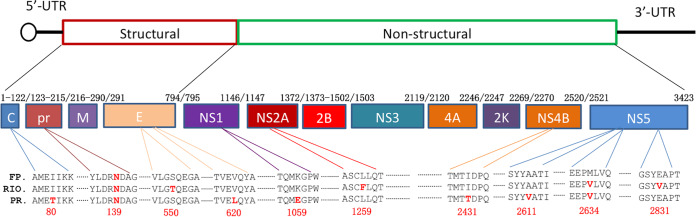
Amino acid variance among Zika virus isolates. The amino acid sequences for the FP (H/PF/2013; GenBank accession no. AHZ13508), PR (PRVABC59; GenBank accession no. AYI50388), and Brazilian isolate (RIO-U1/2016; GenBank accession no. AMD16557) complete polyproteins were aligned using the Clustal Omega algorithm in Geneious Prime 2020.0.3. A graphical representation of the ZIKV genome as well as the resulting protein products is shown. Amino acid variances are highlighted in red, with their position in the polyprotein noted by numerical annotation as well as the nonstructural protein where they reside. The S139N mutation in the prM protein is noted in all isolates for reference. UTR, untranslated region.

The present study focused on the time course of the emergence and persistence of ZIKV in the blood, saliva, cervicovaginal wash (CVW) samples, and maternal reproductive and fetal tissues following intravaginal inoculation using ZIKV-containing semen in baboons at midgestation. In addition, we compared the responses to both French Polynesian (FP) and Puerto Rican (PR) ZIKV isolates to assess differences in virulence, tissue tropism, and vertical transfer. A retrospective study of the ZIKV epidemic in French Polynesia (circa 2013) noted that this was the first instance associating ZIKV with microcephaly and CZS (23, 24). The FP isolate differs from the ancestral Asian ZIKV lineage in harboring a mutation in the prM protein (S139N), which has been stably maintained throughout the virus’s dissemination throughout the Americas. This mutation is associated with enhanced infectivity in human neural progenitor cells (NPCs) and yields more significant microcephaly in mice (25). We selected the PR isolate for comparison to the FP isolate since the CDC reported that one in seven children born from women with confirmed or possible ZIKV infection in Puerto Rico had a birth defect or neurodevelopmental abnormality, similar to the rates of CZS reported in Brazil (26). This suggested that possible mutations in the PR ZIKV isolate may contribute to its increased virulence compared to the FP isolate (26). In addition to the S139N mutation, the PR isolate has acquired several additional mutations resulting in amino acid substitutions (Fig. 1). This study targets many gaps in current knowledge about viremia and pregnancy outcome due to ZIKV infection transmitted sexually in a highly relevant nonhuman primate (NHP), the olive baboon, and compares two relevant ZIKV isolates.

## RESULTS

### Description of animal cohorts and experimental outline.

For this study, adult timed-pregnant female olive baboons (*n* = 6) were used. All baboons were infected via vaginal inoculation with a clinically relevant dose (1 × 10^6^ PFU) of the French Polynesian (H/PF/2013) or the Puerto Rican (PRVABC59) ZIKV isolate. Blood, saliva, and cervicovaginal wash samples were collected (Table 1). In the FP cohort, 2/3 dams developed slight to negligible rash on the abdomen and in the inguinal and axillary regions and no conjunctivitis. One FP isolate-infected dam (FP1) presented with moderate rash on the abdomen, in the bilateral axillary and inguinal regions, and mild conjunctivitis. In the PR cohort, 2/3 dams developed slight to negligible rash on the abdomen and in the axillary/inguinal regions, and only 1 dam (PR3) developed slight conjunctivitis. None of the animals showed signs of any other clinical disease. Baboons were euthanized at 28 dpi. Complete blood counts (CBCs) were evaluated for all females using EDTA-anticoagulated whole-blood samples collected on day 0 and subsequent days postinfection (Table 1) (Idexx ProCyte DX hematology analyzer; Idexx Laboratories, ME). Red blood cell (RBC), hemoglobin, and hematocrit numbers did not show any differences pre- and post-ZIKV infection in all females (data not shown). Platelet counts did not change in response to ZIKV infection in any dam (data not shown).

**TABLE 1:** Inoculation and sampling procedures^*a*^

Dam	Procedure(s) on day after initial inoculation							
	−4	0	4	7	11	14	21	28
FP1	Δ	X, O	Δ	X, O	Δ	Δ	Δ	Ω
FP2	Δ	X, O	Δ	X, O	Δ	Δ	Δ	Ω
FP3	Δ	X, O	Δ	X, O	Δ	X, Δ	Δ	Ω
PR1	Δ	X, O	Δ	X, O	Δ	Δ	Δ	Ω
PR2	Δ	X, O	Δ	X, O	Δ	Δ	Δ	Ω
PR3	Δ	X, O	Δ	X, O	Δ	X, Δ	Δ	Ω

a Baboon semen was obtained via electroejaculation. All procedures were performed with IACUC approval. X, vaginal inoculation with ZIKV-infected (1 × 10^6^ PFU) baboon semen (1 ml) containing the French Polynesian (*n* = 3; FP1, FP2, and FP3) or Puerto Rican (*n* = 3; PR1, PR2, and PR3) isolate (inoculations were repeated at 7-day intervals until evidence of viremia was obtained [qPCR]); O, blood and saliva samples; Δ, cervicovaginal wash, blood, and saliva samples; Ω, maternal/fetal tissue, cervicovaginal wash, blood, and saliva samples collected following euthanasia.

### Viral loads postinfection in whole blood and saliva.

**FIG 2:**
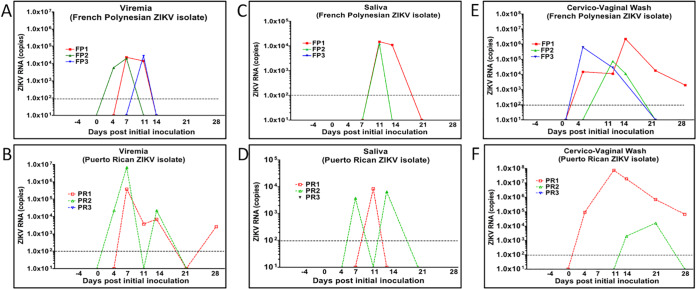
ZIKV RNA (copies per milliliter) in blood (viremia) (A and B), saliva (C and D), or cervicovaginal wash (E and F) samples in midtrimester baboons inoculated intravaginally with baboon semen containing either the FP or PR ZIKV isolate. ZIKV RNA was prepared from specimens collected from each animal at the indicated days postinfection and quantitated by one-step qRT-PCR.

Viral RNA (vRNA) was quantified by one-step reverse transcription-quantitative PCR (qRT-PCR) in RNA extracted from the blood and saliva samples. ZIKV RNA was detected in the blood of all animals infected with the FP isolate of ZIKV and two of the three animals infected with the PR isolate of ZIKV (Fig. 2). Among the dams infected with the FP ZIKV isolate, ZIKV RNA was detected in the blood of all three dams, with dam FP1 being viremic at 7 and 11 dpi, dam FP2 being viremic at 4 and 7 dpi, and dam FP3 being viremic at 4 and 7 dpi (Fig. 2A). Among the dams infected with the PR ZIKV isolate, ZIKV RNA was detected in the blood of 2 of 3 dams: dam PR1 was viremic at 7, 11, 14, and 28 dpi, and dam PR2 was viremic at 4, 7, and 14 dpi. ZIKV RNA was never detected in the blood of dam PR3 at any time point examined (4, 7, 11, 14, 21, 28, or 35 dpi) (Fig. 2B).

In the two dams infected with the FP ZIKV isolate, ZIKV RNA was detected in the saliva at 11 and 14 dpi (FP1) and at 11 dpi (FP2) (Fig. 2C). In the two animals infected with the PR ZIKV isolate, ZIKV RNA was detected in the saliva at 11 dpi (PR1) and at 7 and 14 dpi (PR2) (Fig. 2D).

### ZIKV shedding into cervicovaginal wash samples.

ZIKV RNA was detected in the CVW samples of all three dams infected with the FP isolate of ZIKV and two of the three dams infected with the PR isolate of ZIKV. ZIKV RNA was detected in the CVW samples at 4, 11, 14, 21, and 28 dpi in dam FP1; at 11 and 14 dpi in dam FP2; and at 4 and 14 dpi in dam FP3 (Fig. 2E). In the PR ZIKV-infected dams, two had ZIKV RNA in the CVW, at 4, 11, 14, 21, and 28 dpi for dam PR1 and at 14 and 21 dpi for dam PR2 (Fig. 2F).

### ZIKV RNA in maternal tissues.

In maternal reproductive tissues (uterus, cervix, vagina, and ovaries), ZIKV RNA was not detected in any of the animals inoculated with the FP ZIKV isolate. ZIKV RNA was detected in the uteri of two of the animals inoculated with the PR isolate (dams PR1 and -2) and in the vaginas of two dams (PR1 and -3). ZIKV RNA was present in all of the maternal lymph nodes assessed (axial, mesenteric, and inguinal), except for the mesenteric nodes of dam FP3 and the axial lymph nodes of dam PR3 (Table 2).

**TABLE 2:** ZIKV RNA in maternal reproductive tissues and maternal lymph nodes

Dam	ZIKV RNA level (no. of copies/g)^*a*^						
	Maternal reproductive tissue				Maternal lymph nodes		
	Uterus	Cervix	Vagina	Ovary	Inguinal	Mesenteric	Axial
FP isolate							
FP1	−	−	−	−	2.5E05	3.3E06	1.0E05
FP2	−	−	−	−	7.6E05	2.0E05	5.4E05
FP3	−	−	−	−	7.7E03	−	5.0E03

PR isolate							
PR1	3.2E03	−	6.9E03	−	2.7E03	−	4.1E03
PR2	4.7E04	−	−	−	2.7E05	5.8E05	3.2E05
PR3	−	−	2.8E03	−	1.2E04	7.1E04	−

a −, below the level of detection.

### ZIKV shedding into fetal tissues and placenta.

ZIKV RNA was not detected in any of the fetal tissues (cord blood, cortex, cerebellum, umbilical cord, fetal membranes, spleen, lung, liver, eye, gonads, stomach, intestine, and optic nerve) (data not shown).

Placentas from each dam were sampled from six different locations (different cotyledons). In the animals infected with the FP ZIKV isolate, ZIKV RNA was not detected in any cotyledons sampled. In the animals infected with the PR isolate, ZIKV RNA was detected in two of the animals (PR1 and -2). In one of these animals, ZIKV RNA was detected in five cotyledons sampled (PR1), and in the other animal, ZIKV RNA was detected in four cotyledons sampled (PR2) (Table 3).

**TABLE 3:** ZIKV RNA in placental tissues

Dam	ZIKV RNA level (no. of copies/g) in placental tissue at site^*a*^:					
	1	2	3	4	5	6
FP isolate						
FP1	−	−	−	−	−	−
FP2	−	−	−	−	−	−
FP3	−	−	−	−	−	−

PR isolate						
PR1	2.0E05	1.7E05	−	1.7E05	1.6E05	7.9E04
PR2	−	7.5E04	3.6E05	−	3.6E03	6.8E03
PR3	−	−	−	−	−	−

a −, below the level of detection.

### ZIKV antibody response.

**FIG 3:**
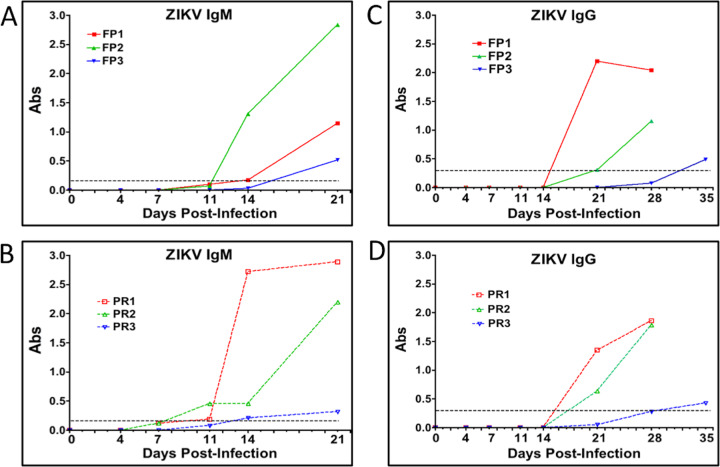
Serum ZIKV IgM (A and B) and IgG (C and D) at the indicated days postinfection in midtrimester baboons inoculated intravaginally with baboon semen containing either the FP or PR ZIKV isolate. The dashed lines represent the assay cutoff controls for IgM and IgG detection in each specific ELISA. The absorbance was read at 450 nm for IgM and at 405 nm for IgG antibodies (Abs) in serum.

ZIKV IgM was detected in sera of 2 of 3 FP ZIKV isolate-infected dams (FP1 and -2) on day 14 and in sera of dam FP3 by day 21 postinfection (Fig. 3A). ZIKV IgM was detected in 2 of 3 PR isolate-infected dams (PR1 and -2) by day 11 postinfection and in dam PR3 on day 18 postinfection (Fig. 3B). ZIKV IgG was detected in the sera of dams FP1 and -2 on day 21 and in the sera of dam FP3 by day 35 postinfection (Fig. 3C). In the PR isolate cohort, dams PR1 and -2 had detectable ZIKV IgG in sera on day 21 postinfection, and dam PR3 had detectable ZIKV IgG on day 32 postinfection (Fig. 3D).

### Immunohistochemistry. (i) Cervix.

**FIG 4:**
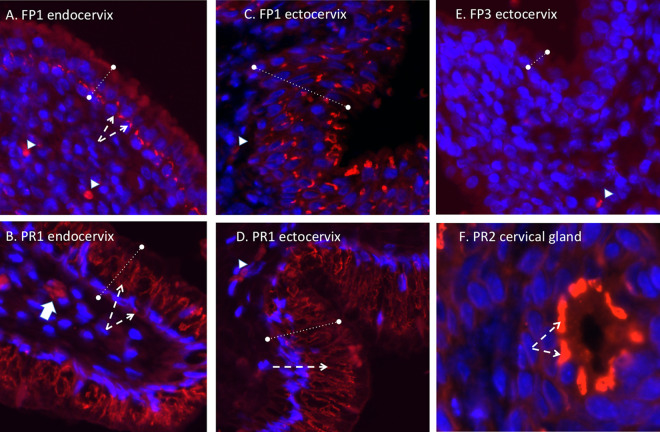
Panflavivirus immunofluorescence (IF) (red, panflavivirus; blue, DAPI) in the cervix. (A to D) In the endocervix (A and B) (upper cervix), ZIKV IF (dashed white arrows) was localized in the epithelium (denoted by the dashed lines) in both the endocervical region (A and B) (simple columnar epithelium) and the ectocervix (C and D) (lower cervical-vaginal region; stratified squamous epithelium) in both PR and FP isolate-inoculated dams. In the FP isolate-inoculated dam (FP1), intense ZIKV IF was observed at the interface of the epithelium-stroma junction in the endocervical region (A and B), with a broader distribution in the epithelium in the ectocervical region (C and D). In the PR isolate-infected dams (B and D), more intense ZIKV IF staining was noted in the epithelium of both cervical regions as well as in occasional cells in the stroma (B, white arrow). (F) In one PR isolate-infected dam, ZIKV IF was also noted in the cervical glands (dashed white arrows). (E) For comparison, a dam infected with the FP isolate that did not exhibit ZIKV IF in either cervical region is shown. Small arrowheads denote autofluorescing red blood cells.

ZIKV immunofluorescence (IF) (panflavivirus) was observed in the cervix of one dam infected with the French Polynesian ZIKV isolate (FP1) but not in dam FP2 or FP3. In dam FP1, the ZIKV IF pattern was localized to the epithelial layer in both the endocervical (upper cervix) and ectocervical (lower cervix, exterior os) regions. In the simple columnar epithelium characteristic of the endocervix, the strongest IF resided near the basil lamina separating the epithelium and stromal layers, with diffuse IF observed in the epithelial layer (Fig. 4A). In the ectocervix, the stratified squamous epithelium exhibited ZIKV IF in cells throughout the epithelium, from the surface to the basal lamina cell layer. No ZIKV IF was noted in the cervical stroma of this dam. ZIKV IF was observed in the cervices of 3/3 dams infected with the Puerto Rican ZIKV isolate (Fig. 4). In these dams, ZIKV IF was observed in the epithelial layers of both the endo- and ectocervices, with more intense IF than in the dam infected with the French Polynesian isolate. In addition, occasional cells exhibited ZIKV IF in the cervical stroma, potentially representing either isolated stromal cells or invading immune cells such as macrophages or neutrophils. In addition, an occasional cervical gland was observed to exhibit ZIKV IF (Fig. 4).

**FIG 5:**
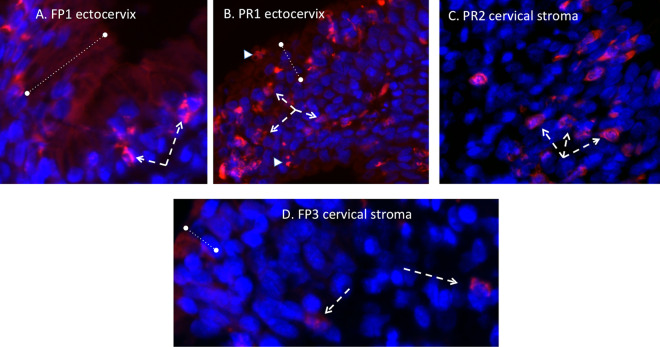
Immunofluorescence staining in the cervix for macrophages (red) (blue, DAPI). (A) In both FP isolate- and PR isolate-infected dams, abundant macrophages were observed, and in the FP isolate-infected dam that exhibited ZIKV IF (FP1), macrophages (dashed arrows) were primarily localized at or near the epithelium-stroma interface. (B and C) In the PR isolate-infected dams that were ZIKV IF positive, macrophages were localized both at the epithelium-stroma interface region (B) as well as deeper in the stromal tissue (C). (D) In dams that exhibited no ZIKV IF in the cervix, only occasional macrophages were noted, typically in the deeper stromal layer.

In the cervix of the French Polynesian and Puerto Rican ZIKV isolate-infected dams that exhibited cervical ZIKV IF, macrophages were frequently observed in the stromal layer juxtaposed to the epithelial layer (Fig. 5). Puerto Rican isolate-infected dams (PR1 and -2) exhibited a greater infiltration of macrophages into the stroma than did dam FP1, with macrophages being observed both adjacent to the epithelial layer as well as deeper in the stromal layer (Fig. 5C). In the French Polynesian isolate-infected dams that did not exhibit cervical ZIKV IF, only occasional macrophages were observed in the stroma (Fig. 5D).

### (ii) Uterus.

**FIG 6:**
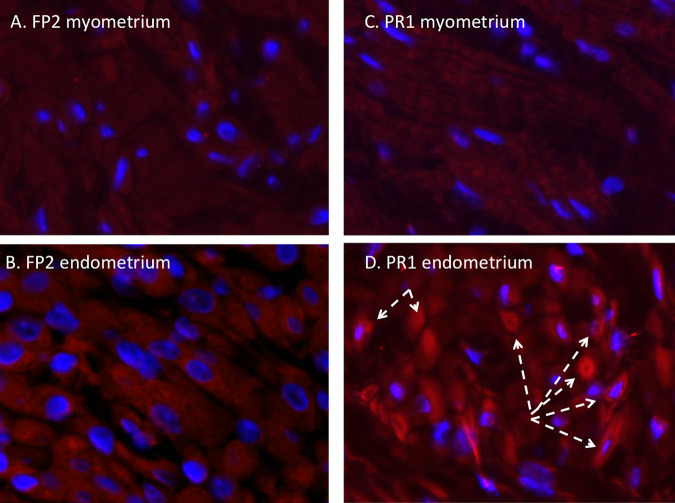
Immunofluorescence staining in the uterus for ZIKV (red) (blue, DAPI). (A and B) ZIKV IF was not observed in the uterus, in either the endometrium or myometrium, of FP isolate-infected dams. (C and D) In the PR isolate-infected dams, ZIKV IF was not observed in the myometrium and was occasionally observed in clusters of endometrial cells (D, dashed arrows).

No Zika virus (panflavivirus) IF was observed in the myometrium of any dam infected with either the French Polynesian or the Puerto Rican ZIKV isolate (Fig. 6). Occasional clusters of ZIKV IF-positive cells were observed in the endometria of the two dams infected with the Puerto Rican isolate that were ZIKV RNA positive in the uterus but not in the dams infected with the French Polynesian isolate or the one dam infected with the Puerto Rican isolate that did not have detectable ZIKV RNA in the uterus. We did not detect macrophages in any of the uterine sections examined from any animal.

### (iii) Placenta and fetal membranes.

**FIG 7:**
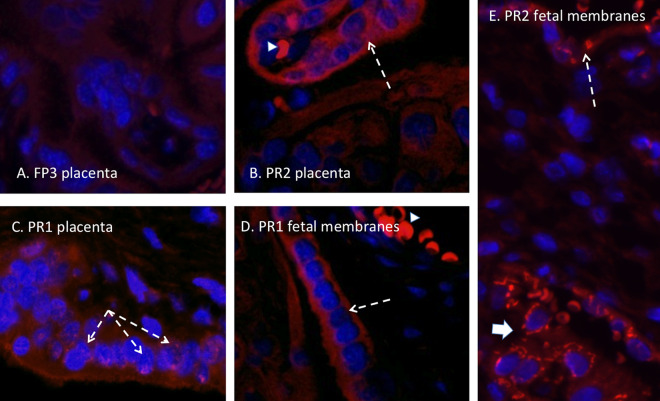
(A) Panflavivirus IF (red, flavivirus; blue, DAPI) staining in the placenta of a ZIKV RNA-negative dam infected with the FP isolate demonstrating a lack of ZIKV IF in the villous placenta. (B and C) ZIKV IF was noted in occasional villous trophoblasts in placentas from dams infected with the PR isolate (white arrows), consistent with ZIKV infection of the syncytial layer. (D) Infection of the amnion epithelium was also observed (arrow). (E) ZIKV IF was also observed in the chorion/decidual layer of the fetal membranes in a dam infected with the PR isolate (large arrow). Autofluorescing red blood cells are indicated with arrowheads.

**FIG 8:**
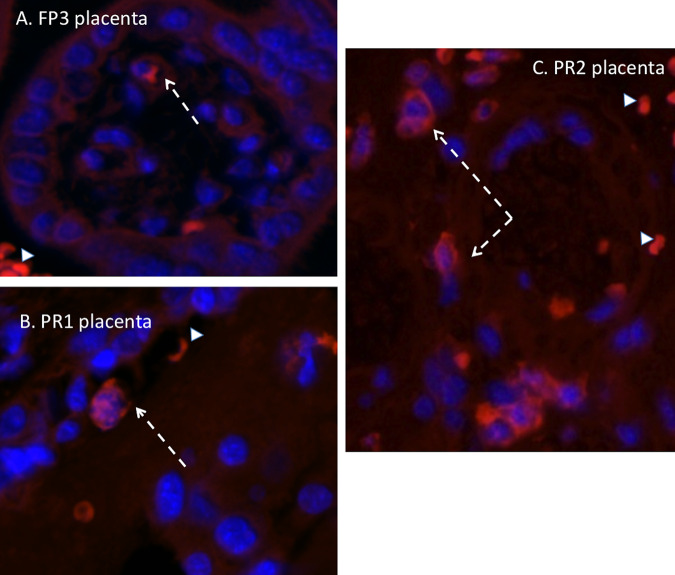
Immunofluorescence staining in the placenta for macrophages (red) (blue, DAPI). (A) In both FP isolate-infected dams, only an occasional macrophage was observed in the maternal and fetal compartments of the placenta (arrow). (B and C) In the PR isolate-infected dams that were ZIKV IF and RNA positive, macrophages were more abundant, particularly in the maternal compartment of the placenta (C, arrow). Arrowheads denote autofluorescing red blood cells.

The dams infected with the French Polynesian ZIKV isolate were negative for ZIKV IF in the placenta and fetal membranes. ZIKV IF (panflavivirus) in the placentas of dams PR1 and PR2 (both positive for placental ZIKV RNA) demonstrated the presence of ZIKV, localized primarily in the syncytial layer with regions exhibiting greater intensity (Fig. 7). In these two dams, we observed ZIKV IF in the amnion epithelium (PR1) and in both the amnion and chorion/decidua of the fetal membranes. Of note, the fetal membranes examined in this study were adjacent to the uteroplacental interface and not the more distal membranes. We did not observe macrophage IF in these sections (Fig. 8).

## DISCUSSION

In the present study, we describe successful ZIKV infection of six timed-pregnant olive baboons at midgestation following vaginal deposition of baboon semen containing either FP or PR isolates of ZIKV. Unlike a study by Müller et al. (20) that demonstrated that human semen inhibited ZIKV infection *in vitro* in cells as well as in human anogenital and reproductive tract tissue explants and various human reproductive tissue explants, we observed highly efficient infection by ZIKV via vaginal inoculation using baboon semen as the carrier. Several studies in mice have also shown sexual transmission of ZIKV through mating of ZIKV-infected male mice with ZIKV-naive female mice and *in utero* transmission in pregnant mice due to sexual transmission (27). It is possible that the vaginal environment disrupts any inhibitory activity of semen in ZIKV infectivity observed in an *in vitro* environment.

We chose multiple inoculations (at 7-day intervals) to mimic probable repeat intercourse in human couples. Similar to our previous study where midgestation pregnant baboons were subcutaneously inoculated with an FP isolate of ZIKV, viremia was observed in the FP isolate-infected dams within 4 to 11 days after the initial inoculation and resolved by 14 dpi (28). However, while viremia in two of the PR isolate-infected dams similarly developed at 4 to 7 dpi, both dams exhibited apparent resolution only to have viremia reoccur, as late as 28 dpi. Surprisingly, one PR isolate-infected dam did not exhibit viremia at any sampling time point yet clearly was infected since an adaptive immune response was observed, along with the detection of ZIKV RNA in various tissues. We can speculate that the reemergence of or prolonged viremia in the PR isolate-infected dams could be attributed to several reservoirs where ZIKV has been found to persist (23, 28–31). We found persistence of ZIKV RNA in both reproductive tissues as well as lymph nodes at the studies’ termination. In rhesus macaques, ZIKV RNA has been detected in the lymph nodes for 5 to 6 weeks postinfection (29). The presence of ZIKV in the lymph nodes is also one possible route of disseminating the virus from the vagina to the cervix, the uterus, and, ultimately, the placenta. Additionally, ZIKV RNA was detected for a prolonged period (up to 4 weeks) in the CVW samples of dams infected with either the PR (two dams) or FP (three dams) isolate. The longer presence of vRNA in CVW samples could also contribute to the reemergence of viremia observed in two of the PR isolate-infected dams. However, it is noteworthy that the FP isolate-infected dams did not show a reemergence of viremia. This may be related to differences in the infectivities of the two isolates. However, at this time, we do not know if the ZIKV found in the saliva, blood, lymph node, reproductive tissue, or cervicovaginal wash samples at the termination of the study was infectious virus.

Studies in pregnant macaques have consistently found prolonged viremia, lasting for or reemerging several weeks to months following subcutaneous inoculation using various ZIKV isolates, including FP, PR, and Brazilian (BR) isolates (32–34). It has been proposed that prolonged or reemergent viremia in macaques could be from tissues harboring ZIKV, including placenta (35). In humans, viremia lasting up to 53 days has been reported (36), but more typically, it is short, lasting 3 to 7 days (37). However, we cannot discount the possibility that we could have seen extended or recurring viremia in the two PR isolate-infected dams had we extended the study for a longer duration. The presence of ZIKV RNA in reproductive tissues and the placenta at the termination of the study suggests that if these tissues harbored infectious virus, then reemergence was possible at later points of gestation. It is also of potential interest that the two dams inoculated with the PR isolate had viremia levels 1 to 2 orders of magnitude higher than those of dams inoculated with the FP isolate. Whether this can be attributed to a greater capacity for infectivity by the PR isolate or simply variability in a small-cohort study remains to be determined.

Vaginal infection with the FP and PR isolates resulted in negligible clinical signs of infection, with only slight to negligible rash, little or no conjunctivitis, and no indication of decreased appetite or water intake or signs of fever or arthralgia. This lack of presentation of clinical signs after vaginal inoculation of ZIKV differed from the moderate to severe clinical signs that we observed in all baboons (primarily rash and conjunctivitis) that we infected subcutaneously with the FP isolate in previous studies of midgestation pregnant baboons (23) as well as nonpregnant female baboons (23) and male baboons (16, 23). The difference in the presentation of clinical signs is likely due to the different routes of ZIKV inoculation (subcutaneous versus vaginal) and is of significant clinical importance since unlike subcutaneous infection, ZIKV infection via sexual intercourse may be more asymptomatic than usual and therefore may evade preventive measures, which could preclude diagnosis, particularly in pregnancy.

We observed ZIKV IF in the cervix of one FP isolate-inoculated dam and all three PR isolate-inoculated dams. For both isolates, ZIKV IF was localized to the epithelial layer of the cervix in both endo- and ectocervical regions. The strongest ZIKV IF intensity was localized at the basal aspect of the epithelial cells at the junction with the stromal layer, which consists primarily of fibroblasts and smooth muscle cells. Despite positive ZIKV IF in one FP isolate-infected dam and two PR isolate-infected dams, none of the dams had ZIKV RNA levels above the limit of detection (although we detected ZIKV RNA in the vaginas of two PR isolate-infected dams). It is possible that our inability to detect ZIKV RNA in the cervix despite ZIKV IF is related to its restriction to the epithelial layer and not the stroma. Our tissues sampled for RNA analysis of the cervix were typically stromal and not epithelial. Alternatively, since in the cervix, the majority of the tissue is stromal, with the epithelium representing a small contribution to the total RNA, the ZIKV RNA level was below the exclusion limit of detection (1 × 10^2^ copies). While no FP isolate-infected dams had ZIKV RNA in the vagina despite being present in the CVW samples, two PR isolate-infected dams had detectable ZIKV RNA in the vaginal tissue. It is noteworthy that the vagina, which is continuous with the cervix, differs from the cervix in that it has a large stratified squamous epithelium that may help harbor the virus. It is possible that the infected epithelium is actively shedding along with the virus in the vagina, thus contributing to its presence in the CVW samples. ZIKV (RNA and protein) was also noted in the uteri of two PR isolate-infected dams, along with positive ZIKV IF staining in the fetal membranes and placentas of the same two PR isolate-infected dams, suggesting the possible spread of ZIKV to the fetus *per se* had the study been for a longer duration. The presence of ZIKV in different reproductive tissues in the PR isolate-infected dams suggests that the reproductive organs in baboons may harbor ZIKV during the acute phase of ZIKV infection through sexual transmission.

The recruitment of macrophages into the cervical stroma has been described during late gestation and was proposed to play an essential role in the remodeling of cervical stroma tissue, which is essential for cervical ripening in preparation for parturition (38). The cervix is referred to as the “gatekeeper” of pregnancy, and as such, premature recruitment of immune cells into the cervix in response to lower-reproductive-tract infection has been proposed to induce the premature loss of cervical integrity, playing a key role in preterm birth. Cervical macrophage infiltration has been well reported in preterm and term cervices in human and animal models (39). Abortion and preterm birth are well described in response to ZIKV in humans and NHPs, including baboons, as we described previously (28). Macrophages can induce cervical connective tissue remodeling via their expression of matrix metalloproteinases (MMPs) and various other factors that help in the breakdown of collagen and junction proteins, resulting in the loss of cervical epithelial integrity required for cervical ripening (40). Macrophages were observed in the FP isolate-infected dam exhibiting cervical ZIKV IF as well as in the cervical ZIKV IF-positive PR isolate-infected dams. In the FP isolate-infected dam, cervical macrophages were observed in the stromal tissue immediately adjacent to the epithelium, indicating monocyte recruitment in response to the virus itself or from a local inflammatory reaction in response to ZIKV infection of epithelial cells. In the PR isolate-infected dams, macrophages were observed adjacent to the epithelial layer as well as deeper in the stromal tissue, indicating the potential breakdown of the epithelium-stroma barrier and entry of the virus into the stromal tissue. In contrast, dams with no cervical ZIKV IF staining had only occasional macrophages in the stromal layer, typically scattered throughout the stroma. It is possible that the recruitment of macrophages due to ZIKV infection of the cervix may induce the breakdown of epithelial cell barrier and integrity similar to that observed during cervical ripening at term. This breakdown could lead to viral access to adjacent reproductive tissues such as the uterus and placenta but also, more importantly, fetal membranes, which lie at the top of the cervical canal and, thus, the amniotic sac and fluid and, ultimately, the fetal compartment, thus exposing the fetus to Zika virus infection.

Vertical transfer of ZIKV in macaques appears to be very efficient, being described to occur at a rate of nearly 100% following infection using various isolates of ZIKV, including FP (34, 35), PR (30, 32, 41), BR (31, 42), and RIO (43, 44) isolates. While we observed no placental infection in the FP isolate-inoculated pregnant dams, vertical transfer to the placenta was observed (by both RNA and IF) in two of the three animals infected with the PR isolate (Fig. 5). In these two animals (dams PR1 and -2), ZIKV RNA was detected in multiple cotyledons, indicating widespread targeting of the placenta. Similar to our previous study infecting dams with the FP isolate (28), ZIKV IF was noted in trophoblast cells. Since the one PR isolate-infected dam without placental ZIKV targeting was also the dam with no noted viremia and latent ZIKV RNA detection in the CVW, this animal may have been infected by a later inoculation. It is clear that dams PR1 and -2 were infected at the first inoculation based on viremia, exhibited ZIKV RNA in the uterus, and had prolonged ZIKV in the CVW. These dams also had notable ZIKV IF in the fetal membranes, indicating that breakthrough of the virus through the placental barrier may have occurred since the membranes used for IF were at the uterus-placenta interface or possibly via a loss of cervical integrity leading to the opening of the cervical canal and access to the membranes. Despite the detection of ZIKV RNA in the placentas of PR isolate-inoculated dams, there was no evidence of vertical transfer of the virus to any of the fetal compartments in any of the animals inoculated. The route of ZIKV infection, subcutaneous versus vaginal, may affect the rate and frequency of vertical transfer to the fetus *per se*. Considering the prolonged presence of virus in the CVW samples, the presence of ZIKV RNA and IF in placental trophoblasts and fetal membranes, and the unanticipated reemergence of viremia (prolonged viremia) in these two PR isolate-inoculated dams, it is possible that vertical transfer would have occurred in these baboons at a later period. Further studies are needed to monitor intravaginally ZIKV-infected pregnant baboons for longer periods postinfection to better understand the fetal outcome of delayed viremia and potential reemergence from immune-privileged sites harboring ZIKV, such as the lymph nodes.

With regard to the adaptive immune response to ZIKV infection, all six of the baboons inoculated with ZIKV developed ZIKV-specific IgM and IgG responses. IgM production following ZIKV infection was noted in all animals at various times, which indicated that the maternal immune system had access to the virus despite the lack of viremia in one of the three animals inoculated with the PR isolate. While IgG titers were detected in all the dams at 21 dpi, this response was either too slow or inadequate to prevent the spread of the virus to various reproductive tissues. It is noteworthy that the IgM and IgG responses were also delayed in the dam inoculated with the FP isolate that displayed delayed viremia (11 dpi; FP3), being observed initially at 21 dpi (IgM) and 35 dpi (IgG). Also, it is noteworthy that the one PR isolate-infected dam that did not exhibit viremia had similar weak IgM (21 dpi) and IgG (35 dpi) responses, with the immune cells likely being exposed to ZIKV via lymph nodes. It is unclear if these dams would have developed a robust neutralizing IgG response if examined at later times postinoculation. As such, we can only speculate that some instances of sexual transmission of ZIKV may not result in a robust, neutralizing adaptive immune response.

While we acknowledge the small animal numbers in our study comparing PR and FP ZIKV isolates, there are clear indications that the PR isolate was more virulent than the FP isolate in terms of the level of viremia, reemergence of viremia, targeting of reproductive tissues, and, importantly, infection of the placenta and the high potential for vertical transfer to the fetus *per se*. Few studies to date have focused on the effect of accumulated mutations in the virus acquired in the Americas compared to either the ancestral Asian isolate or the French Polynesian isolate that acquired the noted S139N substitution in the prM protein. Brazilian isolates, most notably the RIO isolate (RIO-U1/2016), acquired additional substitutions, and the PR isolate differs in several other residues, some in common with the RIO isolate (Fig. 8). How these mutations may have increased virulence remains equivocal. The increased virulence noted here for the Puerto Rican strain (PRVABC59) compared to the isolate from French Polynesia (H/FP/2013) could be interpreted as a difference in the modulation of the host response. Alignment of the complete polyproteins from H/PF/2013, the Brazilian RIO isolate, and PRVABC59 revealed specific amino acid changes in the C protein (I80T), the envelope protein (V620L), and the nonstructural proteins NS1 (K1959E), NS4B (I2431T), and NS5 (A2611V and M2634V) in the PR isolate compared to the FP isolate. Of these, the NS5 mutation is shared with the Brazilian (RIO) isolate. NS1 and NS5 play important roles in flavivirus replication but have also been implicated in the modulation of the host response through interactions with a variety of host proteins (45–47). Targeting of the interferon response by NS1 and NS5 has been documented for multiple flaviviruses, including ZIKV (48–50), and the inhibition of IFN-β by NS1 can be mapped to a specific amino acid residue (45). Considering this, along with the multiple and complex ways in which flavivirus nonstructural proteins manipulate the host response, the amino acid changes identified in NS1 and NS5 could account for the increase in virulence of PRVABC59 through altered interactions with host cellular proteins. However, further investigation is necessary to confirm this hypothesis. In addition, domain III of the flavivirus envelope protein participates in receptor recognition and contains linear epitopes recognized by neutralizing antibodies (51). Interestingly, the single-amino-acid change in the envelope protein of PRVABC59 resides in domain III (V620L) (Fig. 8). However, the two residues are highly similar hydrophobic amino acids, and it is likely that the change is of no consequence to viral pathogenicity.

In conclusion, this study further clarified the transmission of ZIKV following intravaginal inoculation during pregnancy in a novel nonhuman primate model. This important translational model not only more closely recapitulates the course of observed infection patterns in humans but also offers a novel comparator of the infectivities of two contemporary ZIKV isolates. The FP isolate, which was more rarely associated with vertical transmission than the PR isolate, appears to follow the same pattern within this study, which is logical given that ZIKV has continued to mutate during its passage from French Polynesia to the Americas. Future studies, some of which our laboratory is currently undertaking, can focus on the long-term outcomes of ZIKV infection following vaginal inoculation during early gestation and midgestation in pregnancies that are allowed to continue gestation until term. This would be of value as the sexual transmission of flaviviruses such as ZIKV may allow viral persistence or transmission to geographic locales when mosquito transmission is less likely, such as in the winter season. It is also a potential mechanism by which ZIKV can be spread to a population naive to ZIKV infection. Additionally, the course of ZIKV infection following sexual transmission and its consequences to the fetus appear different from those of subcutaneous ZIKV infection, and what this means for the developing fetus and vaccine development is yet to be elucidated. This knowledge may help develop guidelines, preventative measures, and therapeutics to protect against sexual transmission of ZIKV.

## MATERIALS AND METHODS

### Ethics statement.

All experiments utilizing baboons were performed in compliance with guidelines established by the Animal Welfare Act for housing and care of laboratory animals as well as the U.S. National Institutes of Health Office of Laboratory Animal Welfare *Public Health Service Policy on Humane Care and Use of Laboratory Animals* (52) in Association for Assessment and Accreditation of Laboratory Animal Care (AAALAC) International- and National-accredited laboratories. All experiments were conducted in accordance with and with approval from the University of Oklahoma Health Sciences Center (OUHSC) Institutional Animal Care and Use Committee (IACUC) (protocol no. 101523-16-039-I). All studies with ZIKV infection were performed in AAALAC International-accredited animal biosafety level 2 (ABSL2) containment facilities at the OUHSC. Baboons were fed standard monkey chow twice daily and received daily food supplements (fruits). Appropriate measures were utilized to reduce potential distress, pain, and discomfort. Ketamine (10 mg/kg of body weight) was used to sedate baboons during all procedures, which were performed by trained personnel. All animals received environmental enrichment. ZIKV-infected animals were caged separately but within visual and auditory contact of other baboons to promote social behavior and alleviate stress. At the designated times postinoculation, the animals were euthanized according to the recommendations of the American Veterinary Medical Association (2013 panel on euthanasia).

### Animals.

Adult timed-pregnant female olive baboons (*n* = 6) were utilized for this study. All females were multiparous, with a history of successful prior pregnancies. All dams used in this study were determined to be seronegative for West Nile virus (WNV) (23).

### Virus stocks, infection, and sample collection.

Viral stocks of the ZIKV FP isolate (H/PF/2013) and the PR isolate (PRVABC59) were prepared by inoculation onto a monolayer of African green monkey kidney cells (Vero; ATCC CCL-81) with one round of amplification. We have previously reported the use of the FP isolate prepared in our laboratory as described above (23, 28). Single harvests with titers of the FP isolate of 9.67 × 10^7^ PFU/ml and of the PR isolate of 2.27 × 10^8^ PFU/ml were used for vaginal inoculations. The FP isolate (H/PF/2013) was obtained from Helen Lazear (UNC Chapel Hill, Chapel Hill, NC) and was used previously by our laboratory for subcutaneous inoculations in baboons (16, 23, 28). The PR isolate (PRVABC59) was obtained from the ATCC (ATCC VR1843). Animals were anesthetized with an intramuscular dose of ketamine (10 mg/kg) before all procedures (viral inoculation and blood, salivary swab, and cervicovaginal wash sample collection). Timed-pregnant female baboons were infected vaginally with a dose of 10^6^ focus-forming units (FFU) (1-ml volume per dose) of baboon semen spiked with the French Polynesian ZIKV isolate (H/PF/2013) or the Puerto Rican ZIKV isolate (PRVABC59) placed at the cervical os using a speculum to aid with deposition. Semen samples were collected by rectal probe ejaculation, as previously described by our laboratory (16), from a total of 11 male baboons seronegative for WNV and ZIKV and stored immediately at −80°C. Each semen aliquot was thawed on ice before adding virus to it for vaginal infection. Semen samples were chosen at random per inoculation. Inoculations were repeated every 7 days until ZIKV RNA was detected in whole blood by quantitative PCR (qPCR) or, in one dam that never became viremic, through once weekly inoculations. The dosage used to infect the animals in our study is based on previous work done in mosquitos carrying WNV and dengue virus (DENV), where it was estimated that mosquitos carry 1 × 10^4^ to 1 × 10^6^ PFU of the virus (53), from a study evaluating Brazilian ZIKV in a bite from an Aedes aegypti mosquito (54) and from a study of mosquito transmission of ZIKV in rhesus monkeys (55). The pregnant females were infected near midgestation (between 86 and 95 days of gestation [dG]; term is approximately 181 dG). Maternal blood and saliva samples were obtained on the day of inoculation (day 0) as well as at 4, 7, 11, 14, 21, and 28 days postinfection (dpi). Cervicovaginal washes were completed by utilizing 3 ml of normal saline loaded into a 5-ml syringe with a catheter tip and then injected onto the cervix and posterior vaginal fornix and subsequently recollected. These samples were obtained on days −4 (prewash), 4, 11, 14, 21, and 28 postinfection. Ultrasound evaluation of fetal viability was completed with each inoculation and interinoculation specimen collection. Whole blood was collected into EDTA tubes. Saliva and vaginal samples were collected by using a cotton roll Salivette. The sampling procedure for each dam is detailed in Table 1.

At the end of the study for each animal, dams were sedated with ketamine, all maternal samples as well as ultrasound measurements were obtained, and the animals were then rapidly euthanized. A cesarean section (C-section) was quickly performed, cord blood was obtained, and the fetus was euthanized with Euthasol. Maternal and fetal tissues were rapidly collected, and samples were both fixed with 4% paraformaldehyde and frozen on dry ice (stored at −80°C) for each tissue.

### Complete blood counts.

Complete blood counts (CBCs) were obtained for all females from EDTA-anticoagulated whole-blood samples collected on day 0 and subsequent days postinfection, as shown in the experimental timeline (Idexx ProCyte DX hematology analyzer; Idexx Laboratories, ME). CBCs included analyses for red blood cell (RBC), hemoglobin, hematocrit, and platelet counts.

### One-step reverse transcription-quantitative PCR.

Primers and probes used for qRT-PCR were designed as described previously (23, 28, 36). RNA was isolated from maternal and fetal tissues (Tables 2 and 3) using a QIAamp cador pathogen minikit (Qiagen, Valencia, CA). ZIKV RNA was quantitated by one-step quantitative real-time reverse transcription-PCR using a QuantiTect probe RT-PCR kit (Qiagen) on an iCycler instrument (Bio-Rad). Primers and probes were used at concentrations of 0.4 μM and 0.2 μM, respectively, and cycling conditions used were 50°C for 30 min and 95°C for 15 min followed by 40 cycles of 94°C for 15 s and 60°C for 1 min. The concentration of viral RNA (copies per milliliter) was determined by interpolation onto a standard curve of six 10-fold serial dilutions (10^6^ to 10^1^ copies/ml) of a synthetic ZIKV RNA fragment available commercially from the ATCC (ATCC VR-3252SD). The cutoff for the limit of detection of ZIKV RNA was 1 × 10^2^ copies.

### ZIKV ELISA.

ZIKV-specific IgM and IgG antibody responses were assessed in serum samples using commercially available anti-ZIKV IgM (catalog no. ab213327; Abcam, Cambridge, MA) and IgG (catalog no. Sp856C; XpressBio, Frederick, MD) enzyme-linked immunosorbent assay (ELISA) kits. Briefly, 1:100 (for IgM) and 1:50 (for IgG) serum dilutions were performed in duplicate and added to precoated plates available in the kits. The assays were performed according to the manufacturer’s instructions, and the assay was read at 450 nm for IgM and at 405 nm for IgG antibodies in serum.

### Immunofluorescence.

For IF, slides were baked for 1 h at 56°C and deparaffinized, and antigen retrieval was performed in a Retriever 2100 instrument with R-Universal epitope recovery buffer (catalog no. 62719-10, lot no. 180314). After retrieval, slides were blocked in 5% normal donkey serum for 1 h, and primary antibodies in 0.5% normal serum were then added and incubated overnight, humidified, at 4°C (MAC-387, macrophage antibody [Abcam, MA], and anti-panflavivirus antibody [Millipore, CA]). The next morning, slides were removed from incubation at 4°C and allowed to equilibrate to room temperature (RT), covered, on the benchtop for 1 h. Slides were rinsed 4 times for 5 min each with phosphate-buffered saline (PBS), and secondary antibodies were then added and incubated for 1 h, covered, at RT. Donkey anti-mouse IgG F(ab′)_2_ Alexa Fluor 594 (Jackson Immunolabs) was used as a secondary antibody. Slides were rinsed in PBS, counterstained for 5 min with 4′,6-diamidino-2-phenylindole (DAPI) in PBS, and coverslipped using Shur/Mount. Cover glasses were sealed with nail polish, and slides were stored at 4°C and visualized using a fluorescence microscope (Olympus BX43). Images were captured using CellSens imaging software (Olympus).
